# In Situ Antimicrobial Properties of Sabinene Hydrate, a Secondary Plant Metabolite

**DOI:** 10.3390/molecules29174252

**Published:** 2024-09-07

**Authors:** Asta Judžentienė, Dalė Pečiulytė, Irena Nedveckytė

**Affiliations:** Institute of Biosciences, Life Sciences Center, Vilnius University, Saulėtekio Avenue 7, LT-10257 Vilnius, Lithuaniairena.nedveckyte@gf.vu.lt (I.N.)

**Keywords:** sabinene hydrate, fungi associated with *Ips typographus*, Gram-positive and Gram-negative bacteria, fungi, yeasts, inhibition

## Abstract

The objective of this research was to investigate natural products for their potential against pathogenic microorganisms. Sabinene hydrate (SH), a monoterpenoid, is synthesised by numerous different plants as a secondary metabolite. At present, there is a lack of definite investigations regarding the antimicrobial activity of SH itself and its different isomers. The antimicrobial effects of commercially available SH (composed mainly of *trans*-isomer) were evaluated within a range of concentrations in three types of contact tests: solid and vapor diffusion and the macro-broth dilution method. Moreover, the effects of SH on the rate of linear growth and spore germination were also examined. Ethanolic SH solutions were tested against an array of microorganisms, including blue-stain fungi (*Ceratocystis polonica*, *Ophiostoma bicolor*, *O*. *penicillatum*), frequently originating from bark beetle galleries; three fungal strains (*Musicillium theobromae*, *Plectosphaerella cucumerina*, and *Trichoderma* sp.) isolated from a sapwood underneath bark beetle galleries (*Ips typographus*) on spruce (*Picea abies*) stems; *Verticillium fungicola*, isolated from diseased *I. typographus* larvae; two Gram-positive bacteria (*Bacillus subtilis* and *Staphylococcus aureus*), two Gram-negative bacteria (*Escherichia coli* and *Pseudomonas aeruginosa*); five yeasts (*Candida albicans*, *C. krusei*, *C. parapsilosis*, *Saccharomyces cerevisiae*, and *Rhodotorula muscilaginosa*), and two saprophytic fungi (*Aspergillus niger* and *Penicillium notatum*). In solid agar disc diffusion tests, Gram-positive bacteria exhibited greater susceptibility to SH than Gram-negative bacteria, followed by yeasts and fungi. The most resistant to SH in both the disc diffusion and broth macro-dilution methods were *P. aeruginosa*, *A. niger*, and *Trichoderma* sp. strains. Blue-stain fungi and fungi isolated from the *Picea* sapwood were the most resistant among the fungal strains tested. The minimum inhibition concentrations (MICs) generated by SH and determined using a disc volatilization method were dependent on the fungal species and played an important role in the development of microorganism inhibition. The two Gram-positive bacteria, *B. subtilis* and *S. aureus* (whose MICs were 0.0312 and 0.0625 mg/mL, respectively), were the organisms most susceptible to SH, followed by the Gram-negative bacterium, *E. coli* (MIC = 0.125 mg/mL) and two yeasts, *C. albicans* and *C. kruei* (MIC was 0.125 mg/mL and 0.25 mg/mL, respectively). *C. parapsilosis* (MIC = 0.75 mg/mL) was the yeast most resistant to SH. The investigation of antimicrobial properties of plant secondary metabolites is important for the development of a new generation of fungicides.

## 1. Introduction

Sabinene hydrate (SH, also known as 4-thujanol) is a volatile organic compound and belongs to the class of bicyclic monoterpene alcohols. Biosynthesis of monoterpenes occurs mainly by the 2-methyl-erythritol-4-phosphate pathway in plant cell plastids, and geranyl/neryl diphosphate is a key intermediary in the formation of cyclic monoterpenoids [1]. It is important to note that in many cases, the enzymatic synthesis of SH isomers occurs without the formation of free intermediates, e.g., via sabinene or α-thujene [2]. The role of metal ions (such as Mn^2+^ or Mg^2+^) as catalysts is very important for the biosynthesis of SH in plants [2,3]. SH could be in *cis* or *trans* form, it has optical isomers [2,4], and different enzymes are responsible for the biosynthesis of (1*S*,2*R*,4*S*)-(*Z*)-sabinene hydrate and (1*S*,2*S*,4*R*)-(*E*)-sabinene hydrate [4]. This secondary metabolite occurs naturally in a large number of plants and their essential oils (EOs), including *Citrus* [5,6], *Salvia* [7], *Myristica* [8,9,10], *Origanum* [11,12,13,14,15,16,17,18,19,20,21,22], *Thymus* [3,23,24,25,26,27,28], *Mentha* [22,29], *Juniperus* [30,31] and other species. It has been observed that the presence of terpinen-4-ol in the mature leaves and in the steam distillates of *Melaleuca argentea*, *M. dissitiflora*, and *M. linariifolia* is related to high levels of *cis*- and *trans*-sabinene hydrate in the young leaves. Isomers of SH serve as the precursors of terpinen-4-ol, which is formed through non-enzymatic chemical conversion [32]. A significant number of EOs containing appreciable quantities of SH isomers have demonstrated bioactive properties, including strong antifungal and antibacterial activities [12,18,20,28,29,33,34,35,36,37,38,39,40]. A valuable study was conducted to investigate the cultivable bacterial communities of *Origanum* species, from which EOs containing high amounts of SH were obtained [41]. EOs were found to be rich in *trans*-sabinene hydrate (24.0% and 30.8% for *O. vulgare* ssp. *vulgare* and *O. vulgare* ssp. *hirtum*, respectively), and a high degree of biodiversity was found in the bacterial endophytic microbiome. A hypothesis has been proposed that the composition of the EOs may be involved in the formation of microbial communities and that bacteria may be able to colonize the plants by resisting the antimicrobial activity exhibited by the EOs themselves and/or by using some compounds as carbon and energy sources. The composition of microbial communities of plants can impact or modify the EOs’ composition [41].

Plants produce volatile organic compounds for many reasons, and one of them is as a defensive activity against pests and their associated fungi. Terpenoids can be involved in the systemic resistance mechanisms of plants [42,43,44]. It has been reported how the profile of main terpenoids and enantiomeric ratio of major monoterpenes changed in the stem bark of Norway spruce (*Picea abies*) trees inoculated with blue-stain fungus *Ceratocystis polonica* [45]. However, it has also been found that various fungal strains can degrade tree defence metabolites (phenolic compounds) and use them as a carbon source [46,47,48]. Furthermore, the different strains of *Endoconidiophora*, *Ophiostoma* or *Grosmannia* associated with the bark beetle *Ips typographus* Linnaeus (Coleoptera, Curculionidae: Scolytinae) can metabolize monoterpenes in Norway spruce resin, producing volatiles with attractant properties [49]. Bornyl acetate can be metabolized into camphor and α- and β-pinene to *trans*-4-thujanol (SH); both monoterpenoids at specific doses attracted the *I. typographus* beetles in the above study. Additionally, it has been discussed how the volatile emissions of conifers may provide clues regarding tree vitality and suitability for *I. typographus* attacks, i.e., to find suitable host trees for the beetles [50]. On other hand, the repellent effects of SH on both sexes of bark beetle *I. typographus* have been evaluated [51,52,53]. *I. typographus* is a common pest of conifers that attacks Norway spruce, a dominant tree species in the European boreal, montane, and sub-alpine forests, as well as Yezo spruce (*Picea jezoensis*) and Sakhalin spruce (*Picea glehnii* (Fr. Schm.) Masters), dominant trees in eastern Asia [54,55,56,57,58]. During periods when the beetles are not active, they breed in felled, fallen, or windthrown trees, i.e., stumps and logs. During outbreaks, they kill healthy trees, which can have a considerable impact on the forest ecosystem and global environment [59,60].

The interaction between plants and herbivores is often not a simple two-way process; rather, it is mediated by microorganisms, resulting in a complex three-way interaction between plants, herbivores, and microorganisms [55]. The bark beetle *I. typographus* carries spores of several phytopathogenic fungi, including *Ceratocystiopsis minuta, Ceratocystis polonica* (Siem.) C. Moreau, *Ophiostoma bicolor* Davidson & Wells, *O. penicillatum* (Grosmann.) Siemaszko (syn., *Grosmannia penicillata*), *O. piceae* and *O. japonicum* [55,56,57,58]. A well-studied example of a three-way, plant–herbivore–microbe interaction is that observed between conifer trees and tree-killing bark beetles, in which fungal pathogens, developed in the phloem and cambium, are thought to facilitate the beetles’ ability to kill trees [57,58,61].

The main objectives of the investigation were to evaluate the activity of SH (composed mainly of *trans-isomer*), a volatile secondary metabolite of plants against pests’ associated microorganisms:

(i) blue-stain fungi: *Ceratocystis polonica*, *Ophiostoma bicolor* and *O*. *penicillatum* (syn. *Grosmannia penicillata*)), frequently originating from bark beetle galleries;
(ii) other fungi associated with *Ips. typographus,* i.e., three fungal strains: *Musicillium theobromae*, *Plectosphaerella cucumerina and Trichoderma* sp., isolated from sapwood underneath bark beetle galleries (*Ips typographus*) on spruce (*Picea abies*) stems;
(iii) *Verticillium fungicola*, isolated from diseased *I. typographus* larvae;
(iv) and to compare SH activity against:
(v) two Gram-positive bacteria (*Bacillus subtilis* and *Staphylococcus aureus*),
(vi) two Gram-negative bacteria (*Escherichia coli* and *Pseudomonas aeruginosa*);
(vii) five yeasts (*Candida albicans*, *C. krusei*, *C. parapsilosis*, *Saccharomyces cerevisiae*, and *Rhodotorula muscilaginosa*),
(viii) two saprophytic fungi (*Aspergillus niger* and *Penicillium notatum*).

As we mentioned above, a significant number of studies have been conducted on the antimicrobial activity of plant EOs containing appreciable amounts of SH. However, to the best of our knowledge, at present there is a definite lack of publications on the antimicrobial properties of SH itself and its different isomers [12].

It must be emphasised that there are no previous reports related to SH’s effects on most of the microorganisms tested in the present research.

## 2. Results

### 2.1. Agar Disc Diffusion Method

The diameters of inhibition zones (IZs), including paper disc diameter (6 mm), are presented in Table 1.

Microbial strains belonging to different groups (bacteria, yeasts, blue-stain, and other fungi) were differently susceptible to SH in the disc diffusion method. SH was more active against Gram-positive bacteria, followed by Gram-negative bacteria and yeasts. Fungi were more resistant than yeasts and bacteria. Blue-stain fungi as well as other fungi showed low susceptibility to SH in the disc diffusion method. The most resistant blue-stain fungus was *O. penicillatum,* with an IZ of only 13.1 ± 0.7 mm even at the highest SH concentration (1000 μg/disc corresponding 100 mg/mL SH concentration). At the lowest SH concentrations (25 μg/disc and 250 μg/disc), *O. penicillatum* (syn. *Grosmannia penicillata*) showed the full resistance to this monoterpene alcohol. Moreover, a stimulation of melanin synthesis by SH was noticeable in fungus cultures. A similar SH resistance level was detected for *C. polonica*. *O. bicolor* was the most sensitive to SH among the blue-stain fungi as well as among all tested fungi. Very low, albeit detectable, growth inhibition was detected even at the lowest SH concentration (125 μg/disc); IZ diameter increased with SH concentration on the paper disc and exceeded 17.2 ± 3.8 mm at 1000 μg/disc SH concentration.

Surprisingly, in this investigation, the saprotrophic fungi and fungi used (whose trophic attribution is not yet determined), isolated from bark beetle galleries, were more resistant to SH than blue-stain fungi were (Table 1). *A*. *niger* was chosen for our treatments due to its versatility and ability to grow well in a variety of media with different parameters. None of the SH concentrations inhibited *A. niger* or *Trichoderma sp.* strains. All these strains were developed on the paper discs impregnated with 125–250 μg/disc SH content. *A. niger* tolerated also at 500 μg/disc SH concentration. Additionally, *A. niger* was found as a fungus whose sporulation and colony pigmentation were stimulated by SH. *P. notatum* was inhibited only at an SH concentration of 1000 μg/disc, and its IZ was very low (7.9 ± 0.6 mm). *P. notatum* was selected for our studies as a fungus that is ubiquitous in its environment and is able to produce biologically active secondary metabolites. *M. theobromae* showed the largest IZ (average 15.4 mm) as well as *P. cucumerina,* which was susceptible to the lower SH concentration (750 μg/disc).

Gram-positive *B. subtilis* was the most sensitive to SH; its average IZ ranged from 7.0 to 33.5 mm with increased SH concentration, followed by *S. aureus* (Gram-positive), whose average IZ values were 28.8 mm at the highest SH concentration (100 mg/mL) (Table 1). None of the SH concentrations tested inhibited Gram-negative bacterium *P. aeruginosa*. This strain as well as the saprotrophic fungi *A. niger* and *Trichoderma* sp. strains were the microorganisms most resistant to SH in the agar disc diffusion test.

### 2.2. Broth Macro-Dilution Method

The minimum inhibitory concentrations (MICs) of SH were determined against four bacterial and five yeast strains (Table 2).

Gram-positive bacteria, *B. subtilis* and *S. aureus*, with MIC values of 0.0312 and 0.0625 mg/mL, respectively, were the organisms most susceptible to SH, followed by the Gram-negative bacterium *E. coli* (MIC = 0.125 mg/mL) and two yeasts, *C. albicans* and *C. kruei* (MIC values were determined to be 0.125 mg/mL and 0.25 mg/mL, respectively). *C. parapsilosis* was the yeast most resistant to SH (MIC = 0.75 mg/mL), while *S. cerevisiae* and *R. muscilaginosa* showed a medium resistance to this monoterpenoid. None of the concentrations of SH inhibited *P. aeruginosa* (Gram-negative) in this investigation.

The MICs induced in the microbial cultures by SH were from 10 to 100 times lower than those induced by the standard antibiotics (Table 2). Strains of *B. subtilis* and *P. aeruginosa* were the most resistant to chloramphenicol (MICs = 62.50 μg/mL). *E. coli* (MIC = 31.25 μg/mL) was less sensitive, followed by *S. aureus*, as the most susceptible to chloramphenicol.

All filamentous fungi tested were more resistant to SH than yeasts and bacteria; however, none of the SH concentrations induced 100% inhibition of the fungi in their early development stages (germination or germ-tube formation rate). The most susceptible blue-stain fungus was *O. bicolour* (Table 3).

Inhibition of spores started from the lowest tested SH concentration (0.0625 mg/mL) and gradually increased with the increased concentration of the monoterpenoid. Spore germination and inhibition of the ophiostomatoid fungi *C. polonica* and *O. penicillatum* also started from the same SH concentration (2.25 μg/mL). However, *O. penicillatum* showed higher susceptibility (higher inhibition percentages) than *C. polonica* at all SH concentrations tested. Conidia of *A. niger* germinated and were resistant even to very high SH concentrations. An average 0.82% and 1.45% inhibition were caused at 7.5 and 10.0 μg/mL SH concentrations, respectively. Surprisingly, very high resistance to SH was shown by the *M. theobromae* and *P. cucumerina* strains isolated from partially decomposed spruce sapwood underneath *I. typographus* galleries.

As in the case of the broth macro-dilution method used for bacteria and yeasts, nystatin-dihydrate (N-D) activity against the fungi tested was significantly higher than SH activity (Table 3). In the broth macro-dilution method, all fungal strains were fully inhibited by N-D at a concentration of 300 μg/mL, and only a small portion of the propagules of four fungi, including *C. polonica* and fungi associated with beetle galleries (*P. cucumerina*, *A. niger*, and *M. theobromae*) survived at an N-D concentration of 150 μg/mL. *C. polonica* was the strain most resistant to N-D among the all fungi tested. The lowest concentration of N-D, causing 26.71 ± 11.14% inhibition, was 37.5 μg/mL. The lowest N-D concentration inhibited spore germination of both *Ophiostoma* species, and the conidium germination of other fungi was 9.375 μg/mL. However, inhibition percentages were low and insignificant due to the high dispersal of the data.

### 2.3. Sabinene Hydrate Volatilization from Paper Disc (Vapour Phase Activity Test)

**Figure 1:**
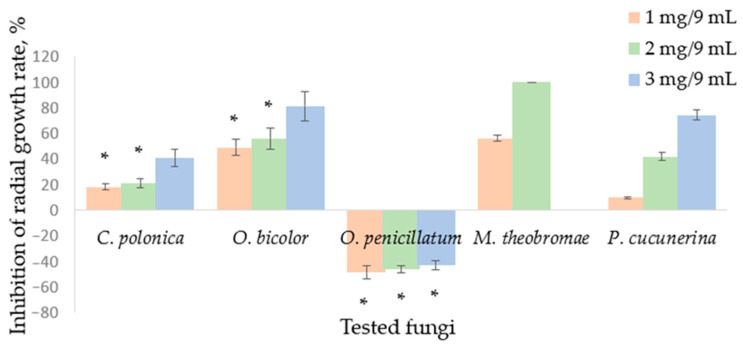
Inhibition of the radial growth rate of fungi by sabinene hydrate supplied in the vapor phase: *Ceratocystis polonica*, *Ophiostoma bicolour*, *Ophiostoma penicillatum* (syn. *Grosmannia penicillata*), *Musicillium theobromae*, and *Plectosphaerella cucunerina*. Mean values represent data from three replicate experiments, bar—SD (standard deviation). Unmarked values are significantly different (*p* < 0.05). A lack of statistically significant difference between the experimental data is marked with asterisks (*).

In Petri dishes, the atmosphere created from paper discs containing 1 mg, 2 mg, or 3 mg of SH induced inhibition of the radial growth rate, which was fungus species-dependent (Figure 1). Inhibition of the radial growth (%) of the blue-stain fungi by SH supplied during the vapor phase was contradictory. Rate of radial growth of *C. polonica* and other fungi tested were inhibited by SH vapor, while *O. penicillatum* was stimulated up to 47.57% compared to the control by the vapor phase obtained from 1 mg SH in the 9 cm^3^ Petri dish. Less stimulation was detected with increasing SH content. *C. polonica* was the least susceptible to SH supplied in the vapor phase, followed by *O. bicolor*.

**Figure 2:**
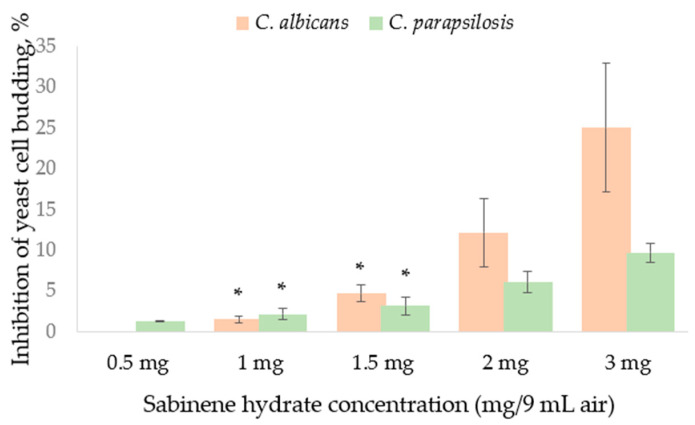
Inhibition of yeast cell budding by the sabinene hydrate supplied in the vapor phase in the disc volatilization method. Mean from three experiments (*n* = 3), bar—SD (standard deviation). Unmarked values are significantly different (*p* < 0.05). A lack of statistically significant difference between the experimental data is marked with asterisks (*).

**Figure 3:**
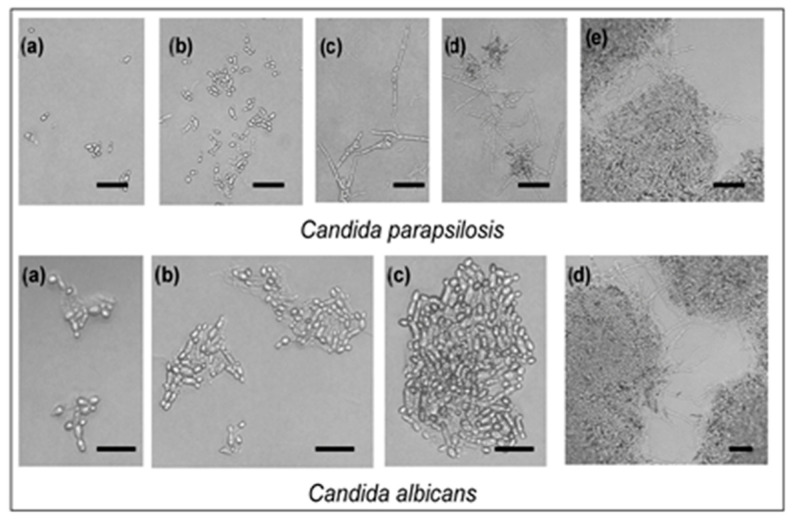
Effect of sabinene hydrate supplied in the vapor phase (volatile fraction evaporating into 9 cm^3^ Petri dish atmosphere from paper disc impregnated with 1 g of this compound) on yeast cell budding and pseudo-mycelium formation. Scale is 10 μm; (**a**–**c**) conidia and conidiophores; (**d**,**e**) conidiophores and conidial masses in preparations. The most representative images were selected from each of the three replicates (*n* = 3).

Two yeasts investigated were also affected by SH supplied in the vapor phase. Neither of the two strains was stimulated by SH in the disc volatilization method (Figure 2). Yeast cell budding as well as pseudo-mycelium formation rate was influenced in the atmosphere containing SH vapor evaporated from 3 mg SH (Figure 2). Greater damage from SH vapor, evaluated as cell budding inhibition percentage (Figure 2), and determined for *C. albicans*. *C. parapsilosis* was more resistant in both cell budding (Figure 2) and pseudo-hypha development (Figure 3).

### 2.4. Results of Dual Culture Tests

Results obtained from dual culture tests differed significantly depending on the culture medium used (malt extract medium (MEA) or malt extract medium amended with spruce phloem extract (MEA + SPE)). Significant differences between the inter-inhibition levels were detected for the blue-stain fungi (Table 4).

**Figure 4:**
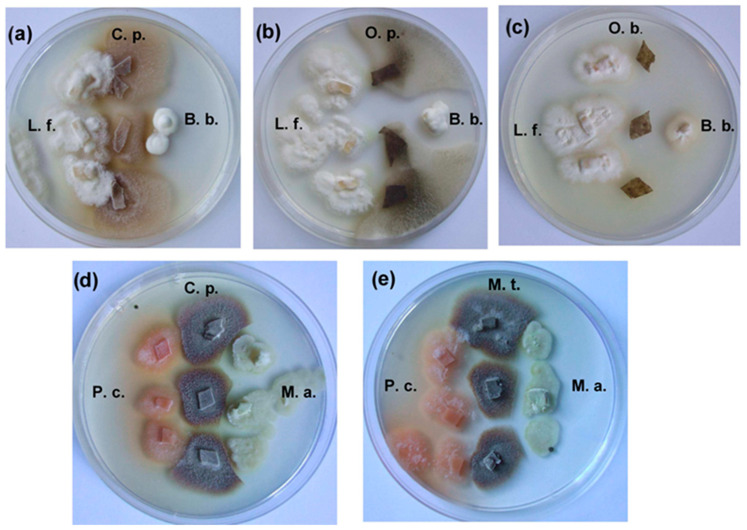
Antagonistic effects of fungi after 10 days’ growth on 2% MEA from (**a**–**e**): C. p.—*Ceratocystis polonica*, O. b.—*Ophiostoma bicolor*, O. p.—*Ophiostoma penicillatum* (syn. *Grosmannia penicillata*), B. b.—*Beauveria bassiana*, L. f.—*Lecanicillium fungicola*, P. c.—*Plectosphaerella cucumerina*, M. t.*—Musicillium theobromae*, and M. a.—*Metarhizium anissopliae*. The most representative images were selected from each of at least three replicates (*n* ≥ 3).

The most active antagonist on the MEA agar medium was *O. bicolor*, whose colonies developed very rapidly and suppressed the growth other ophiostomatoid fungi, especially *C. polonica*. On MEA medium, *O. bicolor* inhibited radial growth of *C. polonica* and *O. penicillatum* by about 99% and 43%, respectively, while *C. polonica* radial growth of both *O. bicolor* and *O. penicillatum* was inhibited by an average of 12.08 and 78.91%, respectively (Table 4). On MEA medium, other fungi studied, including fungicolous *L. fungicola*, entomopathogenic *B. bassiana* and *M. anissopliae*, were resistant to the blue-stain fungi tested and showed high concurrence peculiarities when grown on MEA medium in dual cultures. In this investigation, *L. fungicola* was the most active against the blue-stain fungi. This fungus completely (100%) inhibited growth of the *O. penicillata* and suppressed colony development of *C. polonica* and *O. bicolor* by approximately 82.42% and 38.34%, respectively (Table 4). *O. penicillatum* was the strain most susceptible to *L. fungicola* as well as to fungal entomopathogens, while *O. bicolor* was the most resistant to these fungi on MEA in the dual culture tests. Very strong inhibition of the radial growth rate of the blue-stain fungi was caused by *B. bassiana* and *M. anisopliae*. *B. bassiana* inhibited growth of *C. polonica*, *O. bicolor*, and *O. penicillatum* by an average of 50.77, 22.16, and 94.11%, respectively (Table 4). Sterile zones (3–5 mm wide) were found on MEA in dual *M. anisopliae* and *C. polonica* cultures (Figure 4). The growth inhibition percentage induced by *M. anissopliae* ranged from 17.89 to 62.19% for *O. bicolor* and *O. penicillatum*, respectively (Table 4). Results also confirmed high *P. cucumerina* antagonistic peculiarities against the blue-stain fungi, especially against *O. penicillatum* (~90%). *M. theobromae* was significantly less active; however, its activity markedly increased after two to three weeks of incubation, the when mycelium and sporulation structures of the impacted fungi were intensively degraded (Figure 4). Additional illustrations of the growth of tested fungal colonies are shown in Figures S1–S5 in Supplementary Materials.

In dual culture tests, a different situation was detected when fungal cultures were grown on malt extract medium amended with spruce phloem extract (MEA + SPE). The antagonistic abilities of the *C. polonica* strain changed controversially: *O. bicolor* inhibition increased 4-fold (from 12.08% to 49.31% inhibition), while *C. polonica* partially lost activity against *O. penicillatum* (inhibition activity decreased from 78.91% to 46.77%) (Table 4). Proportionally, the antagonistic activity of *O. bicolor* against *C. polonica* decreased from an average of 98.89 to 24.68% on MEA and MEA + SPE media, respectively. The ability of *O. bicolor* to suppress the radial growth of *O. penicillatum* was almost similar on both culture media. On the MEA + SPE medium, inhibition of *C. polonica* growth rate induced by *O. penicillatum* markedly increased (from 12.43% to 49.27%) (Table 4).

Only *P. cucumerina* kept its antagonistic peculiarities against blue-stain fungi in the dual cultures on MEA + SPE medium. The fungus was stimulated by spruce phloem extracts in MEA medium in control plates. Conversely, *M. theobromae* was inhibited by spruce phloem extracts in the control, and thus its antagonistic peculiarities against blue-stain fungi on this medium decreased by 10–25% as compared with those on MEA medium.

The fungal entomopathogens *B. bassiana* and *M. anissopliae* grew more slowly on the malt extract medium with spruce phloem extract amendments; however, their activity almost did not change in dual culture tests against blue-stain fungi. However, the radial growth rate of the *L. fungicola* strain was stimulated by SH extracts, and its antagonistic abilities against blue-stain fungi increased, with the exception of *O. penicillatum* (100%—inhibition did not change).

## 3. Discussion

Some recent publications concerning bacterial and fungal communities associated with the *Ips typographus* insect have considerably enhanced our understanding of these microbial ecosystems [62,63,64,65]. The bark beetle, its associated fungi, and fungal pathogen invasion are all actually related to the host’s defensive chemistry [66,67,68]. After infection of the host, fungi use a variety of strategies to gain access to the host’s nutrients [69]. Biotrophic fungi acquire nutrients directly from living cells by penetrating them and causing little visible damage [70]. Necrotrophic fungi, in contrast, produce enzymes and toxins to kill plant cells, and then the fungi feed on the nutrients from the dead tissue [71]. Additionally, potential antifungal defense compounds of the plant may contact with necrotrophs [47]. In the case of Norway spruce infestation with *C. polonica*, the plants act by increasing their total terpene content and accumulating monoterpenes with antifungal properties [45,47,61,72,73]. The correlation between terpene content and fungal resistance is strengthened by the data showing that trees that survive *C. polonica* inoculation have much higher terpene concentrations than trees killed by the fungus [73]. This can be explained by several reasons. If microorganisms can improve the fitness of pests by facilitating their nutrient uptake, they must be resistant to terpenes; if the microorganisms reduce the toxicity of host defence compounds (that is, they might be able to transform compounds to be less toxic), they must be resistant to chemical defence.

Our study is the first report concerning the antimicrobial activity of SH (composed mainly of *trans-isomer*) regarding blue-stain fungus and other microorganisms associated with bark beetle *I. typographus* and its development habitat (galleries created in the phloem). Antimicrobial activity of SH against some strains of microorganisms was detected; however, high SH concentrations (up to 500–1000 μg/disc or mL^−1^) were necessary in the broth agar disc diffusion and broth macro-dilution methods. SH was less effective than antibiotics, with a narrow spectrum (affecting only few microorganisms used in the present study as indicators for antimicrobial activity) and with small inhibition zones in the agar diffusion method. SH was found to have low toxicity in the agar diffusion method, whereas it showed high toxicity in the broth macro-dilution method. The reason for this may be that in the broth macro-dilution method, the fungal spores are in direct contact with dissolved SH molecules, which are able to exhibit a greater effect than in the agar diffusion method. As SH is insoluble in water, some re-crystallisation may occur in the medium prepared in water. It is well known that the agar diffusion method has its accuracy impaired as the hydrophobic nature of most EOs or their components prevents the uniform diffusion of these substances through agar medium, which may account for differences in the obtained results.

From the data obtained by the broth macro-dilution method in this study, it can be seen that SH has a significant influence on growth of microbial strains, which is in accordance with the published data regarding EOs containing appreciable amounts of SH [12,20,28,29,34,37,38,39,40].

*B. subtilis* was the most susceptible to all tested SH concentrations, while *P. aeruginosa* was the least susceptible—no SH concentration inhibited its growth. Our results indicated greater susceptibility on the part of the Gram-positive bacteria (*B. subtilis* and *S. aureus*), which has been also reported by other authors regarding EOs containing SH [12,20,36,38].

The weak antibacterial activity against Gram-negative bacteria (*E. coli* and *P. aeruginosa*) can be explained by the presence of an outer membrane. Hydrophilic polysaccharide chains act as a barrier to hydrophobic essential oils and do not allow them to enter and have antibacterial activity. In addition, the resistance of *P. aeruginosa* may be due to its ability to utilise terpenes for its growth [74], or it may be related to the possible resistance genes in plasmids, which can inactivate SH, having antimicrobial potential. High resistance of *P. aeruginosa* against essential oils is well documented [75,76].

An EO from *Origanum majorana*, containing appreciable amounts of both isomers of SH (25.18 and 5.44% of *trans*- and *cis*-, respectively) was assessed against *S. aureus*, and *E. coli* strains; MIC values of 0.125–0.250% and 30–61 µM were obtained, respectively [12]. In the efflux pump inhibitory assay, this EO exhibited substantial activity, especially for the *E. coli* strain. In the case of *S. aureus* strains, the EO and SH exhibited moderate potency on the drug-resistant phenotype. SH was found to be an effective inhibitor of biofilm formation (inhibition 36–86%) in *E. coli* and *S. aureus*, while the EO was ineffective on these strains. In contrast to this, biofilms formed by *E. coli* and *S. aureus* were significantly inhibited by the EO [12]. However, research on the antimicrobial properties of EOs commonly used as condiments in Brazil against *Clostridium perfringens* revealed an MIC = 5.0 mg/mL for marjoram EO with appreciable SH content [38].

Additionally, an antimicrobial activity of EOs with the most characteristic components *trans*-sabinene hydrate and terpinen-4-ol, obtained from several *Origanum* species cultivated in Poland, was evaluated against human respiratory pathogens, such as *S. aureus*, *Haemophilus influenzae*, *H. parainfluenzae*, and *Pseudomonas aeruginosa* [20]. *O. majorana* EO was the most active in the MIC assay and had the highest inhibitory rate in the anti-biofilm assay against all strains in the above study.

In another study [36], a well diffusion assay revealed that EOs from *O. majorana* and *O. vulgare* (containing 10.26 and 27.48% of *cis*-sabinene hydrate, respectively) were active against both the tested Gram-positive, viz., *B. subtilis*, *Micrococcus luteus*, and *S. aureus;* and Gram-negative, viz., *E. coli*, *Klebsiella pneumoniae*, and *P. aeruginosa*, bacteria. MIC values (*v*/*v*) indicated the highest efficacy of *O. majorana* EO against *B. subtilis* (0.5%), *M. luteus* (1%), and *S. aureus* (1%), while *O. vulgare* was most efficient against *E. coli* (2%) and *K. pneumoniae* (2%).

EOs from Tunisian *A. absinthium* L. were tested for their antibacterial and antifungal activity against 10 indicator microorganisms including seven pathogenic bacterial references (*E. coli*, *S. typhimurium*, *S. aureus*, *P. aeruginosa*, *A. hydrophila*, *L. monocytogenes*, and *B. cereus*), and against three fungus species (*A. flavus*, *A. niger* and *C. albicans*); and displayed antimicrobial activity against all tested strains with variable degrees. For Gram-negative bacterial strains, comparable levels of antibacterial activities were observed (MIC range from 12.5 to 25% *v*/*v*) for the oil containing of 11.83% (*Z*)-sabinene hydrate; the highest activity was recorded against the bacteria *P. aeruginosa* [39].

Fungi, especially filamentous fungi, were more resistant to SH than bacteria were. *C. albicans* was the most sensitive yeast, and *A. niger* as well as *Trichoderma* sp. were the most resistant fungi. Blue-stain fungus *O. bicolor* was the most susceptible fungus in both the disc diffusion and broth macro-dilution methods as compare to other blue-stain fungi. The pioneer *Picea* tree tissue invader *C. polonica* was more resistant to SH than *O. bicolor* and *O. penicillatum*. It was determined that spores from the blue-stain fungi were the most susceptible to SH. No inhibition was found in the growing mycelium. Conversely, some stimulatory effects such as increased radial growth and melanin synthesis were observed, especially in the case of *O. penicillatum* cultures (Figure 1).

SH induced changes in the morphology of blue-stain fungi: dominance of one stage with elimination of the other. It must be noted that blue-stain fungi and saprotrophic fungi were in a strong antagonistic relationship in this investigation. Moreover, saprotrophic fungi were also resistant to SH.

Fungal entomopathogens (*Beauveria bassiana*, (Bals.-Criv) Vuill. and *Metarhizium anissopliae* (Metschn.) Sorokin) were among the fungi distributed in *I. typographus* galleries; they were found on the larva cadavers. Thus, it was interesting to determine their relationship with blue-stain fungi in an in situ experiment. As was mentioned above, phytopathogens are well known to alter plant chemistry. However, even though several studies have considered the effects of plant chemistry on insect pathogens, knowledge on how phytopathogenic fungi may influence herbivores’ vulnerability to entomopathogens is almost completely lacking. Contradictory results in studies with fungal entomopathogens have been found [77,78,79]. It has been suggested that this might reflect differences in their dependence on host insects [80,81]. Entomopathogens should be considered important natural enemies of bark beetles, and their importance has been discussed regarding other herbivores [82].

In our study, two fungi, *M. theobromae* and *P. cucumerina*, isolated from the *I. typographus* galleries and their phloem and one strain from the genus *Lecanicillium* W. Gams & Zare [*L. fungicola* (Preaus) Zare & W. Gams], isolated from *I. typographus* cadavers, were resistant to SH and very active antagonists to blue-stain fungi when grown on standard MEA medium (Figure 4). Their antagonistic properties weakened when phloem extract medium was used for the investigation of the dual cultures, suggesting that they are not pioneer sapwood invaders and develop using partially degraded phloem and/or secondary metabolites from other fungi. Despite this, *M. theobromae* and *P. cucumerina* inhibited development of the blue-stain fungi and overgrew their mycelium and sporulation structures (Figure 4). Only a few studies have addressed the question of how a decrease in host plant suitability might affect the efficiency of entomopathogens. *O. majorana* EOs containing SH exhibited strong antifungal properties against rice seed-borne fungi, such as *Bipolaris orzyae*, *Curvularia lunata*, *Fusarium verticilliodies,* and *F. graminearum* [18]. Additionally, *Mentha spicata* EO (content of SH: 7.04%) showed strong antifungal activity to plant-pathogenic fungi, such as *Fusarium oxysporum* f. sp. *radicis-lycopersici* (Sacc.) W.C. Synder & H.N. Hans, *Rhizoctonia solani* J.G. Kuhn. *Alternaria solani*, and *Verticillium dahliae* Kleb; and against selected bacterial strains of *Xanthomonas* spp. [29].

In our investigation, the results of the paired cultures indicated the possibility that they are associated with bark beetle–plant–fungal pathogen systems. If they inhibit the growth of blue-stain fungi, which are hypothesized as its feeding source, they thus influence the development of insects indirectly through feeding. Some explanation could be found in an investigation conducted by Rostás, M. et al., in which the duration of larval development from hatching to pupation increases by about 9% when *Phaedon cochleariae* (Chrysomelidae) larvae feed upon fungus-infected Chinese cabbage leaves. Higher larva mortality under prolonged development was induced by the entomopathogenic fungus *Metarhizium anisopliae* (Metsch.) Sorok. [83]. *I. typographus* larvae feeding on healthy tree tissues could also survive entomopathogen attacks; while feeding on diseased tree tissues, this ability can be lost. Other factors must be also considered, such as, e.g., the induction of defensive plant compounds by the phytopathogen. The effects of plant allelochemicals influencing insect susceptibility to entomopathogens are known [83]. Artificial diets containing plant secondary metabolites may enhance insect mortality caused by entomopathogens. Thus, knowledge about *I. typographus* fungal pathogens is valuable too, and investigations could be continued in the future.

## 4. Materials and Methods

### 4.1. Sabinene Hydrate (Dissolution and Preparation for Bioassays)

A commercial synthetic SH (analytical standard, ≥97.0% (GC), composed of isomers, containing a predominant quantity of *trans*-sabinene hydrate, Sigma-Aldrich, Merck KGaA, Darmstadt, Germany) was used for the investigations. SH, being insoluble in water, was dissolved in ethanol (96%). Stock solutions of sabinene hydrate [10% (100 mg/mL) or 20% (200 mg/mL)] were prepared, from which half-and-half dilutions in a range of sabinene hydrate concentrations (6.25, 12.50, 25.00, 50.00, 100.00, and 200.0 mg/mL) were used for in vitro treatments against test organisms.

### 4.2. Test Organisms

The selected test organisms used to evaluate the antifungal and antibacterial activity of the sabinene hydrate were as follows:

(a) blue-stain fungi: *Ceratocystis polonica* (Siem.) C. Moreau 1994-169/113, *C. polonica* BIGTC-2133, *Ophiostoma bicolor* Davidson & Wells BIGTC-2133, and *O. penicillatum* (Grosmann.) Siemaszko (syn. *Grosmannia penicillata* (Grosmann) Goid) 2006-209/44/2;
(b) other mycelial fungi, which were isolated from *Ips typographus* Linnaeous (Coleoptera, Curculionidae, Scolytinae) galleries in *Picea abies* L. stemps and from its discolorated sapwood, such as *Musicillium theobromae* (Turconi) Zare & W. Gams (syn. *Stachylidium* *theobromae* Turconi, *Verticillium theobromae* E.W. Mason & S. Hughes BIGTC-20132 and *Plectosphaerella cucumerina* (Lindt.) W. Gams BIGTC-136, *Aspergillus niger* Tiegh. BIGTC-9823, *Penicillium notatum* Wehmer BIGTC-8914, and *Trichoderma* sp. BIGTC-2131;
(c) yeasts *Candida albicans* BIGTC-MK2, *C. parapsilosis* BIGTC-MK9, *C. krusei* BIGTC-MK3 (Blastomycetes, Cryptococcales, Cryptococcaceae) and *Saccharomyces cerevisiae* BIGTC-MK11 (Saccharomycetes, Saccharomycetales, Saccharomycetaceae) phylogenetically related to the Ascomycota, as well as *Rhodotorula muscilaginosa* BIGTC-056 (Urediomycetes, Sporidiales) phylogenetically related to Basidiomycota;
(d) Gram-positive bacteria *Staphylococcus aureus* BIGTC-BK06 and *Bacillus subtilis* BIGTC-BK09;
(e) Gram-negative bacteria *Escherichia coli* BIGTC-BK08 and *Pseudomonas aeruginosa* BIGTC-BK19.

All cultures were obtained from the Microorganism Culture Collection of Nature Research Centre (Vilnius, Lithuania), except three strains, *C. polonica* 1994-169/113, *O. bicolor*, and *G. penicillata* 2006-209/44/2, which were received from the KTH Royal Institute of Technology, Stockholm, Sweden.

As reference compounds, chloramphenicol and nystatin-dihydrate were used for treatments with bacteria and fungi, respectively.

### 4.3. Preparation of Test Organisms

The cultures of test organisms were maintained in agar slants at 4 °C (bacteria in nutrient agar (NA), fungi in 2% malt extract agar (MEA), and yeasts in Sabourad agar (SA); all media were purchased from Liofilchem SRL, Roseto degli Abruzzi (TE), Italy). To obtain stock cultures, fungi were pre-cultured on 2% MEA at 25 °C for four weeks (Ophiostomatoid fungi) and 7 days (other fungi), yeasts—on SA for 3 days, and bacteria—on NA for 2 days. Fungal conidia were taken from the slants using sterile saline (0.9% NaCl, *w*/*v*) containing 0.05% Tween 80. Mycelia were removed by filtration through sterile gauze, and the filtrate was adjusted up to 1–2 × 10^5^ conidia mL^−1^. Bacterial and yeast cells were picked from freshly grown slant cultures using 10 mL of sterile saline, and decimal cell solutions were prepared in the sterile saline up to 10^7^ and 10^6^ colony-forming units (CFU) mL^−1^ (bacteria and yeasts, respectively).

### 4.4. In Vitro Antifungal and Antibacterial Activity Testing

The CLSI (Clinical and Laboratory Standards Institute) method for antimicrobial susceptibility testing was modified for sabinene hydrate testing. We used two preliminary methods: the agar diffusion method for all strains of microorganisms and the disc volatilization method (vapor phase activity) for microbial strains selected as the most sensitive in the agar diffusion method. Additionally, direct SH activities (minimal inhibition concentrations, MICs) against bacteria and yeasts tested were detected by the micro-broth dilution method; additionally, SH direct activity against the fungi was determined. Activity of SH against spore (or conidium) germination was evaluated for bacterial and yeast strains. A standard antibiotics, nystatine-dihydrate (ROTH, Carl Roth GmbH + Co., Karlsruhe, Germany), was used to control the sensitivity of the tested fungi; chloramphenicol (ROTH, Carl Roth GmbH + Co., Germany) was applied to control the sensitivity of the tested bacteria. Concentrations were explored depending on the method used. Turbidity measurements of the medium were not performed.

#### 4.4.1. Agar Disk Diffusion Method

The evaluation of SH activity against test strains of microorganisms was carried out by the disc diffusion method, which is normally used as a preliminary check and to select between different oils and their constituents. Solidified 2% MEA, SA, and NA (for fungi, yeasts, and bacteria, respectively) media were used for the SH activity treatments. Media were inoculated with 100 μL inoculums of appropriate microorganisms (10^7^, 10^6^, and 105 CFU mL^−1^ suspension concentrations of bacteria, yeasts, and fungi respectively) and were spread over the plates using a sterile rod display in order to obtain a uniform microbial growth on both control and test plates. After the inoculums’ absorption by agar, sterile filter discs (Whatman no 1, 5 mm diameter) were impregnated with 10 μL solutions of sabinene hydrate (at 6.25, 12.50, 25.00, 50.00, 100.00, and 200.00 mg/mL concentration in 96% ethanol). These preparations corresponded to SH concentrations of 0.0625, 0.125, 0.25, 0.50, 1.0, and 2.0 mg per disc, respectively. Filter discs moistened with ethanol alcohol were placed on the seeded Petri dish as a negative control. Filter discs impregnated with nystatine-dihydrate (30 mg/mL corresponding 30 μg/disc) for fungi and chloramphenicol (30 mg/mL, corresponding 30 μg/disc) for bacteria were used as a reference control.

All Petri dishes were sealed with sterile laboratory parafilm to avoid the eventual evaporation of SH. The dishes were left for 30 min at room temperature to allow the diffusion of oil, and then were incubated for 24 h for bacteria at 30 °C, for 48 h for yeasts, and 48–98 h for fungi at 25 °C. After the incubation period, the mean diameter of the inhibition halo where the test microorganisms did not grow (clearly visible inhibition zone) or the growth was partly inhibited was measured in millimeters.

#### 4.4.2. Broth Macro-Dilution Tests

The minimal inhibitory concentration (MIC) values were determined for bacterial and yeast strains using the macro-well dilution method as described [84]. Bacterial strains were cultured for 18 h at 30 °C in Müller-Hinton broth (MHB, Liofilchim, Roseto Degli Abruzzi (TE), Italy); yeasts were cultured for 48 h at 25 °C in 2% malt extract broth (MEB, Liofilchem, Italy). Then, test cultures were suspended in sterile saline (0.9% NaCl) and diluted to produce a final density of 2.5 × 10^6^ cfu mL^−1^ for the bacteria and 1.5 × 10^5^ cfu mL^−1^ for the yeasts.

The 24-well plates were prepared by dispensing into each well 490 μL of appropriate nutrient broth. An aliquot (10 μL) of SH, initially prepared at a 10% concentration (100 mg/mL; *w*/*v* ethanol, was added to each of the first wells, followed by 2-fold serial dilution to obtain concentrations in a range from 1.0 to 0.0625 mg/mL *w*/*v* (corresponding from 0.1% to 0.000625% *w*/*v* concentrations). The prepared inoculums of the test cultures were separately added to the wells (500 μL each). The final volume in each well was 1 mL. The last well, containing 500 μL of MHB (or MEB) without test substance and 500 μL of the inoculum, was used as a negative control. Standard antibiotics, tested at concentrations ranging (chloramphenicol) from 50.0 μg/mL to 1.5625 μg/mL and (nystatine-dihydrate) from 30.0 μg/mL to 0.9375 μg/mL, were used as positive controls. Ethanol was not tested because the even highest concentration used in the plate well was equal 0.096 % (10 μL ethanol (96%) per 1 mL well content = 0.096%). Culture medium + test sample of the sabinene hydrate was used as the sterility control. The plates were covered with a sterile plate sealer and incubated at 30 °C (bacteria) and 25 °C (yeasts) for 18–24 h and 48 h, respectively. Growth of the bacteria and yeasts was indicated by the presence of turbidity and/or white fur at the bottom of the well. The minimum inhibition concentrations (MICs) were determined as the lowest concentration preventing visible growth. Additionally, viability was proven by making subcultures from 50 μL from each well showing no turbidity and negative control on nutrient agar (NA; Liofilchem, Italy) or SA medium. For each strain, the growth conditions were checked and plates were incubated as described above.

Fungal strains in the macro-broth dilution method were treated by some modifications of the method. Fungi spore inoculums, corresponding approximately 1.0–1.5 × 10^4^ conidia mL^−1^, were prepared as stocks, from which 0.1 mL aliquots were transferred into each plate well containing either 2% MB medium or 2% MB amended with an appropriate sabinene hydrate concentration. Plates were incubated for 4–8 h, and then 100 mL^−1^ of each well content was sub-cultured for 24–48 h on 2% MEA medium (three replicate plates were used per well). After incubation, the number of growing colonies in the control and SH treated variants was counted. Growth inhibition percentage was calculated by comparing the number of CFUs in the SH-treated culture with that of the control. Spores which did not form colonies were considered as having lost their viability. Microscopic observations were made to determine growth (germination and germ tube length) retardation under SH impact.

#### 4.4.3. Disc Volatilization Method (Vapor Phase Activity)

This method was used to evaluate the activity of SH vapor from impregnated paper discs against the blue-stain fungal strains isolated from *P. abies* sapwood and two fungal strains associated with *I. typographus* galleries, the same strains which were used in the disc diffusion method. Agar plugs of actively growing fungi (blue-stain fungi after 2 weeks and other fungi after 7 days’ growth on the 2% MEA) were placed in the center of the Petri dish (covered with 2% MEA). Different aliquots (10 μL, 20 μL, and 30 μL) of the SH solution were added to 6 mm sterile blank paper discs placed in the center of the cover of the glass Petri dish (6 cm diameter). The dishes were sealed with laboratory parafilm to avoid eventual evaporation of SH followed by incubation. The volume of the dish in which SH vapor was distributed was 9 cm^3^. Blanks were prepared by adding 10 μL of ethanol alcohol to the paper discs. Practically, vapor from either of the SH contents (1 g, 2 g, or 3 g per dish) evaporated into the dish atmosphere above the disc. The effectiveness of SH was calculated as a percentage inhibition of the linear fungus growth (in mm) compare to the control (disc with appropriate content of water). Each assay in these experiments was repeated three times and the results (inhibition %) were expressed as average values ± SD (standard deviation).

#### 4.4.4. Dual Culture Tests

Agar plugs (5 mm diameter) from two actively growing test fungi (blue-stain fungi after 2 weeks and other fungi after 7 days growth on the MEA medium were placed at the different ends of the Petri dish dishes, covered with either of MEA medium or MEA amended with spruce phloem-extract (MEA + SPE)). Dishes were incubated at 25 °C in the dark. The radial growth of mycelium (in mm) was measured daily and compared with that in the MEA and MEA + SPE plates simultaneously inoculated with each fungus only as control.

Percent of test fungus inhibition by the other fungi was evaluated and expressed as calculated percent inhibition (*PI*):

$$PI=\frac{C-T}{C}\times 100$$

where *C* is the growth of the test fungus (mm) in the absence of the other fungi; *T* is the growth of the test fungus (mm) in the presence of the opposite fungus.

### 4.5. Statistical Analysis

The obtained data were statistically processed and expressed as means and standard deviation (SD) values, using the XLSTAT program (trial version, Addinsoft 2014, Paris, France). Convergence of results was based on at least three independent measurements. Evaluation of statistically significant differences between tested parameters was performed by one-way ANOVA statistical analysis. Differences between obtained values were compared using Student’s *t*-test, at a significance level of α = 0.05. IBM SPSS Statistics software (Version 28.0.1.1(15), New York, NY, USA) was applied to calculate the *p*-values. Statistically significant differences were set at *p* values lower than 0.05.

## 5. Conclusions

The current study has made a contribution to knowledge regarding the in situ antimicrobial properties of sabinene hydrate (SH), which is a quite common secondary metabolite in plants. For the first time, the effects of SH (composed mainly of *trans*-isomer) were revealed against the following microorganisms: blue-stain fungi (*Ceratocystis polonica*, *Ophiostoma bicolor*, *O. penicillatum*), frequently originating from bark beetle galleries; three fungal strains (*Musicillium theobromae*, *Plectosphaerella cucumerina*, and *Trichoderma* sp.), isolated from sapwood under bark beetle galleries (*Ips typographus*) on spruce (*Picea abies*) stems; and *Verticillium fungicola*, isolated from diseased *I. typographus* larvae. Additionally, monoterpenoid activity was tested and compared against two saprophytic fungi (*Aspergillus niger* and *Penicillium notatum*), two Gram-positive (*Bacillus subtilis* and *Staphylococcus aureus*), and two Gram-negative bacteria (*Escherichia coli* and *Pseudomonas aeruginosa*); and five yeasts (*Candida albicans*, *C. krusei*, *C. parapsilosis*, *Saccharomyces cerevisiae*, and *Rhodotorula muscilaginosa*). The antimicrobial effects were evaluated over a range of SH concentrations in three types of contact tests, namely solid and vapor diffusion and the macro-broth dilution method. SH was less effective than antibiotics, such as chloramphenicol and nystatin-dihydrate, in the agar diffusion method. SH was found to have low toxicity in the agar diffusion method against the micro-organisms tested, whereas it showed a high toxicity in the broth macro-dilution method. In solid agar disc diffusion tests, Gram-positive bacteria were more susceptible to SH than Gram-negative bacteria, followed by yeasts and fungi. The most resistant to SH in both the disc diffusion and broth macro-dilution methods were *P. aeruginosa*, *A. niger,* and *Trichoderma sp.* strains. Blue-stain fungi and fungi isolated from the *Picea* sapwood were the most resistant among the fungal strains tested. The values of the minimum inhibition concentrations generated by SH and determined using the disc volatilization method depended on the tested fungus species. SH showed a more or less important role in the development of microbial inhibition. The Gram-positive bacteria *B. subtilis* and *S. aureus* were the organisms most susceptible to SH, followed by Gram-negative bacterium, *E. coli* and two yeasts, *C. albicans* and *C. kruei.* The strains of *C. parapsilosis* were the yeasts most resistant to SH. Further research may be focused on evaluating the bioactivity of SH against other pathogenic microorganisms and/or investigation of antimicrobial properties of another isomer (*cis*-) of this monoterpenoid.

The investigation of antimicrobial properties of plant secondary metabolites is important for the development of a new generation of fungicides. In general, this study has provided new comprehension regarding the application of SH as a natural product derived from plants. The use of renewable products offers great prospects for more economical, environmentally friendly, and effective development of industry and agriculture.

## Figures and Tables

**Table 1:** Mean diameters of inhibition zones (mm) of microorganisms using sabinene hydrate in an agar disc diffusion test *.

Microorganisms	Samples						
	Sabinene Hydrate, mg/mL (μg/disc)					ETA ^1^	Reference ^2^
	12.5 (125)	25.0 (250)	50.0 (500)	75.0 (750)	100 (1000)		
Mycelial fungi							
*Ceratocystis polonica*	0.0 ^3^	0.0 ^a^	6.0 ± 0.2 ^a^	13.4 ± 0.5 ^b, c^	15.5 ± 0.5 ^b^	11.5 ± 1.5 ^c^	30.0 ± 3.4
*Ophiostoma bicolor*	7.2 ± 0.3 ^a, b, e^	7.6 ± 0.6 ^a, f^	9.2 ± 0.8 ^b, c, d, g^	12.3 ± 2.1 ^c^	17.2 ± 3.8 ^d^	9.0 ± 0.9 ^e, f, g^	41.9 ± 2.7
*Ophiostoma penicillatum* ^4^	0.0 ^a^	0.0 ^a^	6.0 ± 0.1	7.8 ± 0.6 ^b^	13.1 ± 0.7	10.5 ± 1.5 ^b^	30.5 ± 3.1
*Musicillium theobromae*	0.0	6.0 ± 0.1 ^a, b, d^	6.0 ± 0.1 ^a, c, e^	6.0 ± 0.3 ^b, c, f^	15.4 ± 0.2	6.8 ± 1.3 ^d, e, f^	36.8 ± 2.9
*Plectosphaerella cucumerina*	0.0 ^a^	0.0 ^a^	6.0 ± 0.0	7.4 ± 0.5 ^b^	11.8 ± 0.6 ^c^	7.0 ± 0.3 ^b^	12.6 ± 1.8 ^c^
*Aspergillus niger*	0.0 ^a, b^	0.0 ^a, c^	0.0 ± 0.0 ^b, c^	6.0 ± 0.1 ^d, e^	6.0 ± 0.0 ^d, f^	6.0 ± 0.0 ^e, f^	30.0 ± 1.0
*Penicillium notatum*	0.0 ^a^	0.0 ^a^	6.0 ± 0.0 ^b, c^	6.0 ± 0.0 ^b, d^	7.9 ± 0.6	6.0 ± 0.2 ^c, d^	28.2 ± 2.3
*Trichoderma* sp.	0.0 ^a^	0.0 ^a^	6.0 ± 0.1 ^b, c, e^	6.0 ± 0.0 ^b, d, f^	6.0 ± 0.2 ^c, d, g^	6.5 ± 0.3 ^e, f, g^	30.3 ± 3.5
Yeasts							
*Candida albicans*	6.0 ± 0.1 ^a^	8.2 ± 0.3 ^a^	11.7 ± 1.5	16.1 ± 0.4 ^b^	21.4 ± 0.8 c	10.2 ± 0.9	26.4 ± 6.1 ^b, c^
*Candida krueii*	6.0 ± 0.2 ^a, b, e^	6.0 ± 0.1 ^a, c, f^	8.5 ± 1.8 ^b, d, g^	8.6 ± 0.9 ^d, h^	15.5 ± 0.7	7.1 ± 0.8 ^e, f, g, h^	29.0 ± 2.3
*Candida parapsilosis*	6.0 ± 0.1 ^a, b, c, d^	6.0 ± 0.2 ^a, e^	6.0 ± 0.3 ^b, c, d, f^	7.0 ± 0.0 ^d^	18.4 ± 0.9 ^g^	6.6 ± 0.0 ^d, e, f^	16.8 ± 1.8 ^g^
*Saccharomyces cerevisiae* ^5^	6.0 ± 0.2 ^a, d^	6.0 ± 0.1 ^a, b, c, e^	6.0 ± 0.1 ^b, c, f^	8.1 ± 0.3	18.3 ± 0.6	6.5 ± 0.2 ^d, e, f^	36.9 ± 4.2
*Rhodotorula muscilaginae* ^4^	6.0 ± 0.1 ^a, d^	6.0 ± 0.1 ^a, b, e^	7.0 ± 0.2 ^b, c, f^	9.2 ± 1.3 ^c^	14.4 ± 0.6	8.2 ± 0.4 ^d, e, f^	33.7 ± 3.2
Bacteria							
*Bacillus subtilis*	7.0 ± 0.4 ^a, b^	7.9 ± 0.8 ^a^	14.8 ± 2.3 ^c^	18.9 ± 2.1 ^d^	33.5 ± 2.7 ^e^	6.0 ± 0.3 ^b^	22.4 ± 6.1 ^c, d, e^
*Staphylococcus aureus*	6.0 ± 0.1 ^b^	8.0 ± 0.3 ^a^	9.1 ± 0.7 ^a^	13.4 ± 0.4	28.8 ± 1.6 ^c^	6.0 ± 0.2 ^b^	36.5 ± 4.8 ^c^
*Escherichia coli*	6.0 ± 0.0 ^a^	7.8 ± 0.5 ^a, b, d, e, f^	7.6 ± 0.2 ^c^	8.4 ± 0.9 ^b, c^	23.8 ± 1.2	7.2 ± 0.4 ^d, e, f^	31.7 ± 1.6
*Pseudomonas aeruginosa*	6.0 ± 0.1 ^a, b, d, k^	6.0 ± 0.1 ^a, e, f, g, l^	6.0 ± 0.2 ^b, e, h, i, m^	6.0 ± 0.3 ^c, f, h, j^	6.0 ± 0.4 ^d, g, i, j, n^	7.5 ± 0.7 ^k, l, m, n^	45.7 ± 8.6

* Inhibition zone diameter of 6 mm value is equal to disc diameter, implying no activity. The results are presented as means ± SD (standard deviation) of three replicate tests from three experiments (*n* = 9); paper of disc diameter is included. ^1^ Solvent ethanol as negative control is presented only at the highest concentration (10 μL/disc). ^2^ Reference compounds are chloramphenicol (30 μg/disc) for bacteria and nystatin-dihydrate (30 μg/disc) for fungi. ^3^ Fungus growth on the paper disc. ^4^ Strains whose cultures are stimulated by vapor phase. ^5^ Strains whose cultures are negatively influenced by the vapor phase. Unmarked values are significantly different (*p* < 0.05). For each individual microorganism, obtained values (at different SH concentrations and references) with no significant difference are marked by the same letters (^a, b, c, d, e, f, g, h, i, j, k, l, m, n^) (*p* > 0.05).

**Table 2:** Minimum inhibition concentrations (mg/mL) of SH for microbial strains by the macro-broth dilution method.

Organisms	Sabinene Hydrate, mg/mL (μg/mL)	Nystatin-Dihydrate, μg/mL	Chloramphenicol, μg/mL
Yeasts			
*Candida albicans*	0.125 (125)	9.375	–
*Candida kruei*	0.25 (250)	37.5	–
*Candida parapsilosis*	0.75 (750)	150.0	–
*Saccharomyces cerevisiae*	0.5 (500)	18.75	–
*Rhodotorula muscilaginosa*	0.5 (500)	9.375	–
Gram-positive bacteria			
*Bacillus subtilis*	0.0312 (30)	–	62.50
*Staphylococcus aureus*	0.0625 (62.5)	–	<15.625
Gram-negative bacteria			
*Escherichia coli*	0.125 (125)	–	31.25
*Pseudomonas aeruginosa*	>1.0 mg/mL *	–	62.50

* *P. aeruginosa* was revealed to be resistant to all tested SH concentrations.

**Table 3:** Germination effects (%) of sabinene hydrate on fungi development with the macro-broth dilution method *.

Fungi	Sabinene Hydrate Concentration, mg/mL (μg/mL)					
	0.0625 (62.5)	0.125 (125)	0.25 (2500)	0.5 (500)	0.75 (750)	1.0 (1000)
*Ceratocystis polonica*	0.0 ± 0.0 ^a^	0.0 ± 0.0 ^a^	8.62 ± 4.80 ^b^	17.11 ± 4.35 ^b, c^	31.19 ± 5.17	29.64 ± 7.07 ^c^
*Ophiostoma bicolor*	0.52 ± 0.21 ^a^	1.59 ± 1.43 ^a^	21.21 ± 9.01	47.98 ± 10.09 ^b, c, d^	59.93 ± 19.08 ^b, c, d^	67.35 ± 9.93 ^d^
*O. penicillatum*	0.0 ± 0.0 ^a^	0.0 ± 0.01 ^a^	18.93 ± 7.17	32.14 ± 5.74	49.46 ± 12.38 ^b^	49.21 ± 3.67 ^b^
*Plectosphaerella cucumerina*	0.0 ± 0.0 ^a, b^	0.0 ± 0.01 ^a^	0.0 ± 0.01 ^b^	7.35 ± 2.51 ^c^	7.68 ± 5.22 ^c^	19.89 ± 6.47
*Aspergillus niger*	0.0 ± 0.0 ^a, b^	0.0 ± 0.00 ^a, c^	0.0 ± 0.01 ^b^	0.0 ± 0.00 ^c^	0.82 ± 0.31 ^d^	1.45 ± 0.82 ^d^
*Musicillium theobromae*	0.0 ± 0.0 ^a^	0.0 ± 0.00 ^a^	6.73 ± 2.98 ^b^	13.25 ± 4.91 ^c, d^	8.97 ± 6.14 ^b, c^	24.00 ± 9.45 ^d^
	**Nystatin-Dihydrate (N-D) Concentration, mg/mL (μg/mL)**					
	**0.009375 (9.375)**	**0.01875 (18.75)**	**0.0375 (37.5)**	**0.075 (75)**	**0.15 (150)**	**0.3 (300)**
*C. polonica*	0.0 ± 0.0 ^a^	0.0 ± 0.0 ^a^	26.71 ± 11.14	63.28 ± 24.41	98.55 ± 1.23 ^a^	100 ^a^
*O. bicolor*	0.92 ± 0.55	13.07 ± 4.46	48.92 ± 20.02	80.27 ± 19.22 ^a^	100 ^a, b^	100 ^b^
*O. penicillatum*	0.08 ± 0.03	0.88 ± 0.22	37.52 ± 11.55	96.27 ± 3.71 ^a, b^	100 ^a, b, c^	100 ^c^
*P. cucumerina*	0.19 ± 0.08	14.38 ± 9.71 ^a^	33.61 ± 22.04 ^a^	87.23 ± 10.71 ^b^	99.01 ± 0.04 ^b, c^	100 ^c^
*A. niger*	0.04 ± 0.01	3.56 ± 1.88	22.83 ± 6.47	86.87 ± 5.23 ^a^	86.17 ± 13.64 ^a, b^	100 ^b^
*M. theobromae*	0.62 ± 0.17	31.05 ± 27.91 ^a^	66.37 ± 33.06 ^a, b, c, d^	90.87 ± 6.61 ^b, c, d^	96.14 ± 2.87 ^b, c, d^	100 ^b, c, d^

* Results are presented as an inhibition of spore (or conidium) germination by SH percentages as compared to control. The results are expressed as a mean ± SD (standard deviation) of three agar plates (with emerged colonies) for each of three wells/each SH dilution. Unmarked values are significantly different (*p* < 0.05). For each individual microorganism, values (at different SH and N-D concentrations) with no significant difference are marked with the same letters (^a, b, c, d^) (*p* > 0.05)

**Table 4:** Inhibition (%) of radial growth in dual culture test *.

Inhibiting Fungus	Medium	Inhibition of Radial Growth Rate (%)		
		*C. polonica*	*O. bicolor*	*O. penicillatum*
*Ceratocystis polonica*	MEA		12.08 ± 1.88	78.91 ± 11.47
	MEA + SPE		49.31 ± 10.31 ^a^	46.77 ± 20.28 ^a^
*Oprhiostoma bicolor*	MEA	98.89 ± 1.11		43.28 ± 10.99
	MEA + SPE	24.68 ± 9.55 ^a^		41.63 ± 4.75 ^a^
*Ophiostoma penicillatum*	MEA	12.43 ± 4.88	65.14 ± 22.07	
	MEA + SPE	49.27 ± 10.87 ^a^	66.89 ± 15.68 ^a^	
*Beauveria bassiana*	MEA	50.77 ± 12.32	22.16 ± 3.84	94.11 ± 5.33
	MEA + SPE	45.54 ± 6.27 ^a^	24.02 ± 8.33 ^a^	91.58 ± 8.03
*Lecanicillium fungicola*	MEA	82.42 ± 9.45 ^a^	38.34 ± 11.66	100 ^a^
	MEA + SPE	86.82 ± 13.15 ^a^	56.72 ± 15.21	100 ^a^
*Metarhizium anissopliae*	MEA	58.62 ± 15.27 ^a^	17.89 ± 5.16	62.19 ± 13.04 ^a^
	MEA + SPE	57.44 ± 20.33 ^a^	21.14 ± 8.87	60.73 ± 21.04 ^a^
*Musicillium theobromae*	MEA	59.46 ± 10.19 ^a, b^	78.45 ± 14.37 ^a, c^	46.28 ± 11.17 ^b, c^
	MEA + SPE	32.29 ± 14.03 ^a, b^	53.67 ± 22.02 ^a, c^	36.66 ± 6.04 ^b, c^
*Plectosphaerella cucumerina*	MEA	46.55 ± 5.09	14.56 ± 4.08	90.01 ± 4.17
	MEA + SPE	44.10 ± 16.99 ^a^	18.23 ± 2.78 ^a^	89.65 ± 10.20

* MEA: Malt extract medium, MEA + SPE: Malt extract medium amended with spruce phloem extract. Results were presented as means of three tests ± SD (standard deviation). Unmarked values are significantly different (*p* < 0.05). For each inhibiting fungus, inhibition values (in different medium and type of fungi (*C. polonica*, *O. bicolor* and *O. penicillatum*)) with no significant difference were marked by the same letters (^a, b, c^) (*p* > 0.05).

## Data Availability

Data are contained within the article and Supplementary Materials.

## Supplementary Materials

The following supporting information can be downloaded at https://www.mdpi.com/article/10.3390/molecules29174252/s1, Figure S1: *Ceratocystis polonica*: (a) colonies after 14 days’ growth on 2% MEA medium; (b,d) synnemata in culture; (c,e,f) synnemata and conidia in preparates; (g) micro ascus. Scales are indicated in pictures. Figure S2: *Ophiostoma penicillatum* (syn. *Grosmannia penicillata*): (a–c) colonies after 14 days’ growth on 2% MEA, PDA and SA media, respectively; (d) synnemata in culture; (e) conidiogenesis in culture; (f) synnemata in culture; (g,h) *Leptographium* state; (a–c) scales are 10 μm; other scales are 100 μm. Figure S3: *Musicillium theobromae*: (a) colonies after 10 days’ growth on 2% MEA; (b–d) conidiophores and conidial masses in culture; (e,f) conidiophores and conidial masses (heads) in preparates; (g) aggregates from mycelium in culture; (h,i) conidiophores and conidia. Scales are indicated in pictures. Figure S4: *Plectosphaerella cucumerina*: (a) colonies after 10 days’ growth on 2% MEA; (b–d) hyphae, conidiophores, and conidia in culture; (e,f) conidiophores and conidia in preparates. Scales are indicated in pictures. Figure S5: *Ceratocystis polonica* colonies after 6 days’ growth on: (a) MEA medium, (b) mineral medium without glucose and with phloem extract medium, and (c) mineral medium with phloem. *Ophiostoma penicillatum* (syn. *Grosmannia penicillata*) colonies after 6 days’ growth on: (d) MEA medium, (e) mineral medium without glucose and with phloem extract medium, and (f) mineral medium with phloem.
