# Supplementary material for: In Situ Antimicrobial Properties of Sabinene Hydrate, a Secondary Plant Metabolite
Source: Molecules. 2024 Sep 7;29(17):4252. doi: 10.3390/molecules29174252 (PMC11396819; doi:10.3390/molecules29174252)
Supplement: Supplementary file 1 [file molecules-29-04252-s001.zip › molecules-3162978-supplementary.pdf]

in situ **Antimicrobial Properties of Sabinene Hydrate, a Plant Secondary Metabolite**

Asta Judžentienė*, Dalė Pečiulytė and Irena Nedveckytė

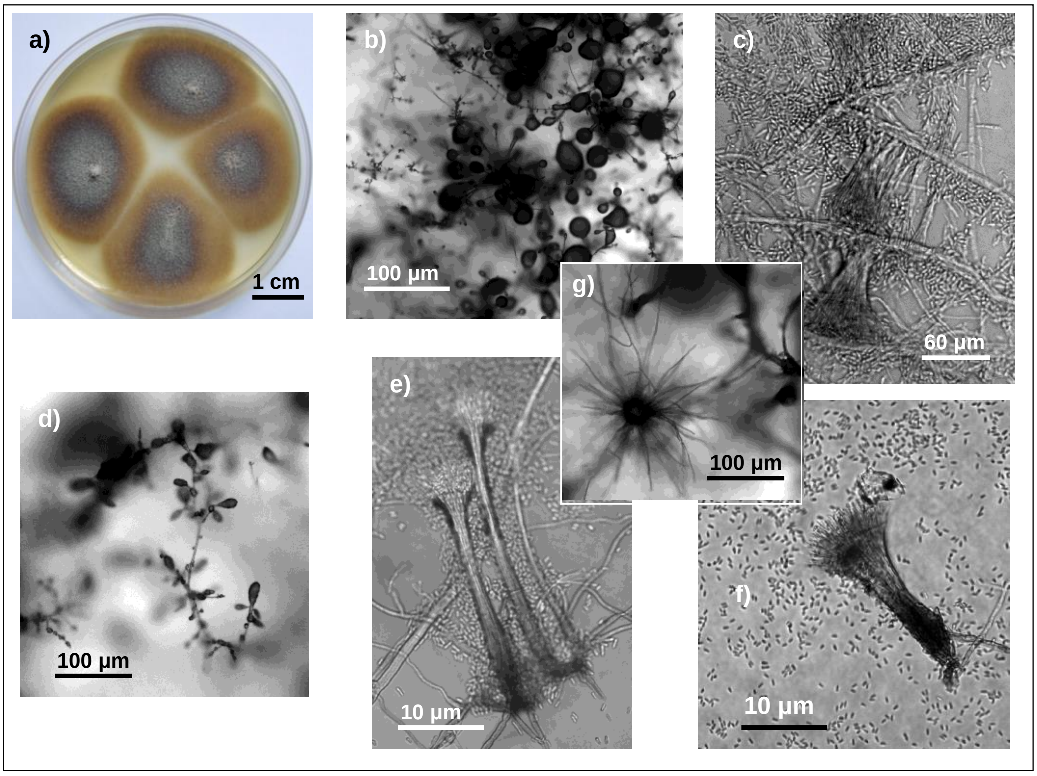

Figure S1. *Ceratocystis polonica*: a) colonies after 14 days growth on 2% MEA medium; b) and d) synnemata in culture; c), e) and f) synnemata and conidia in preparates; g) micro ascus. Scales were indicated on pictures.

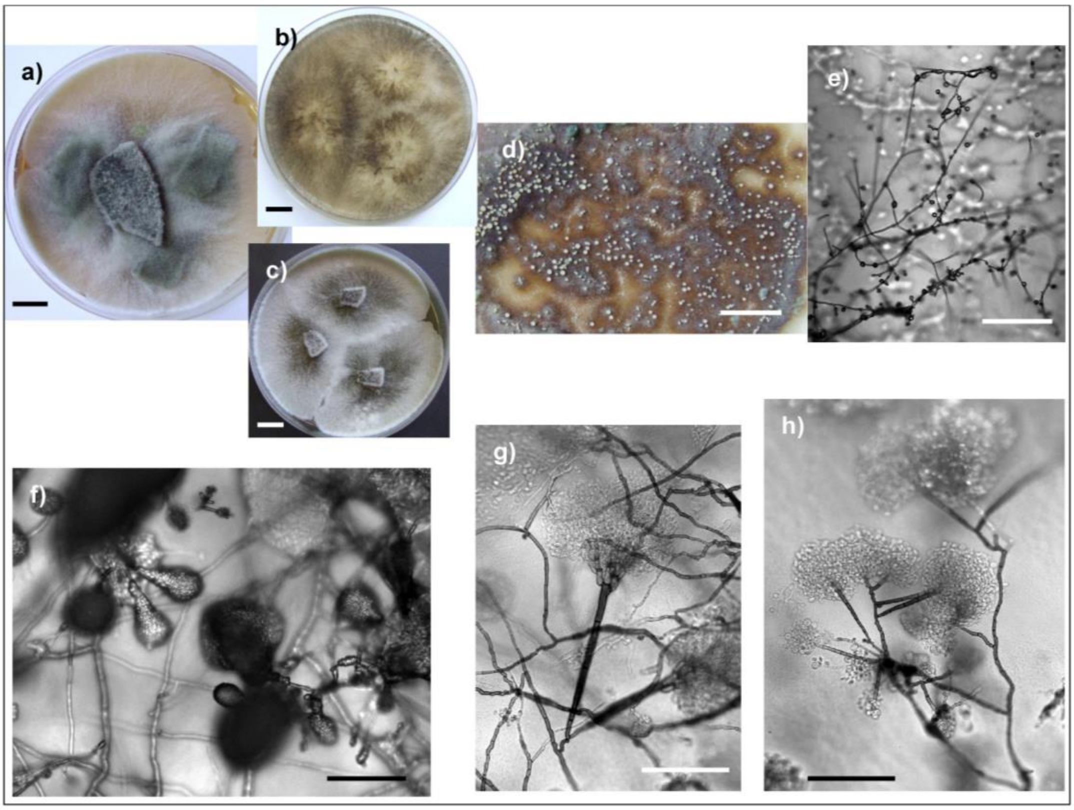

Figure S2. *Ophiostoma penicillatum* (syn. *Grosmannia penicillata*): a), b) and c) colonies after 14 days growth on 2% MEA, PDA and SA media, respectively; d) synnemata in culture; e) conidiogenesis in culture; f) synnemata in culture; g) and h) *Leptographium* state; a), b) and c) scales were 10 μm; other scales were 100 μm.

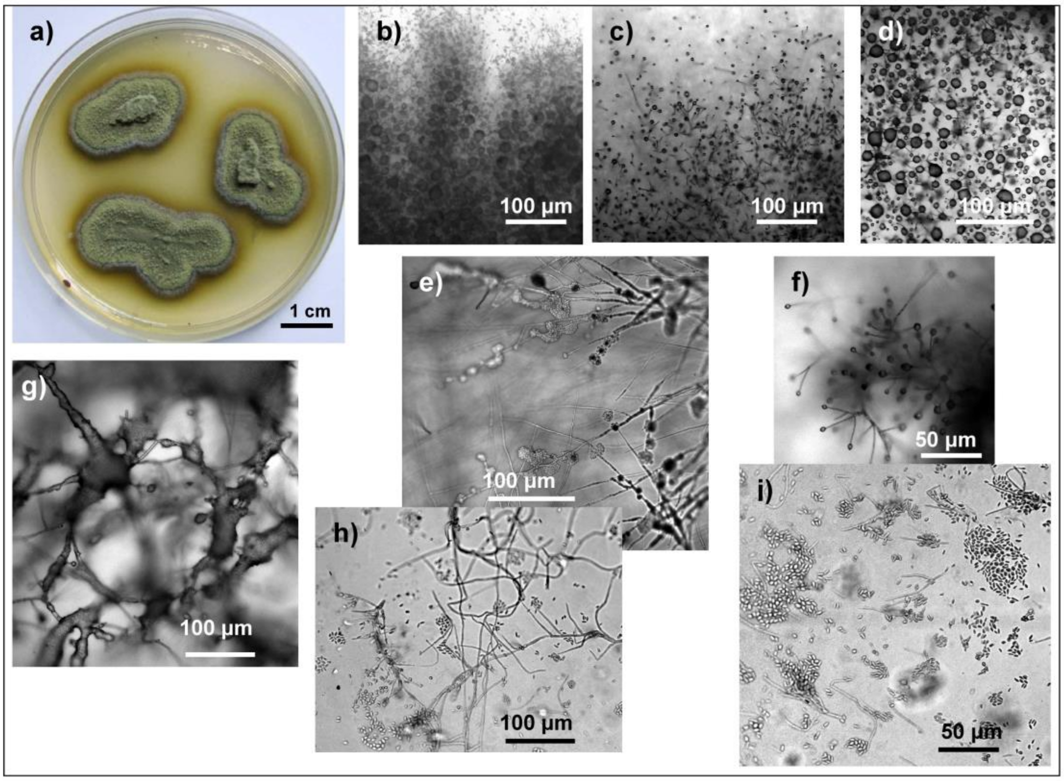

Figure S3. *Musicillium theobromae*: a) colonies after 10 days growth on 2% MEA; b), c) and d) conidiophores and conidial masses in culture; e) and f) conidiophores and conidial masses (heads) in preparates; g) aggregates of mycelium in culture; h) and i) conidiophores and conidia. Scales were indicated on pictures.

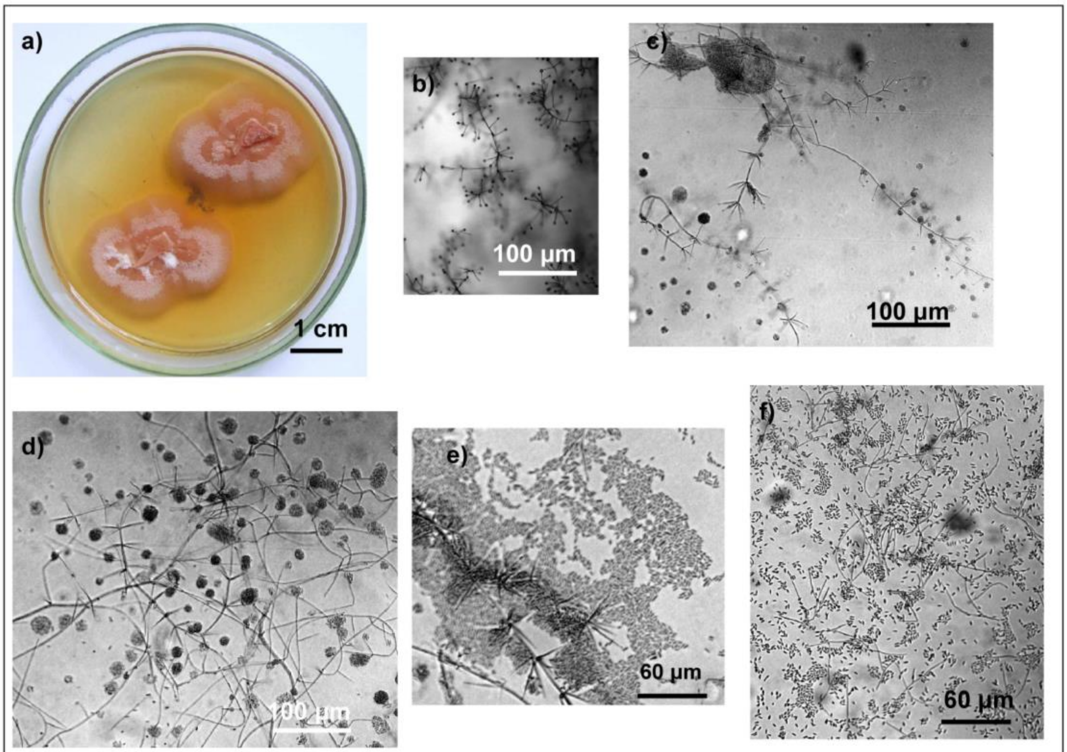

Figure S4. *Plectosphaerella cucumerina*: a) colonies after 10 days growth on 2% MEA; b), c) and d) hyphae, conidiophores and conidia in culture; e) and f) conidiophores and conidia in preaparates. Scales were indicated on pictures.

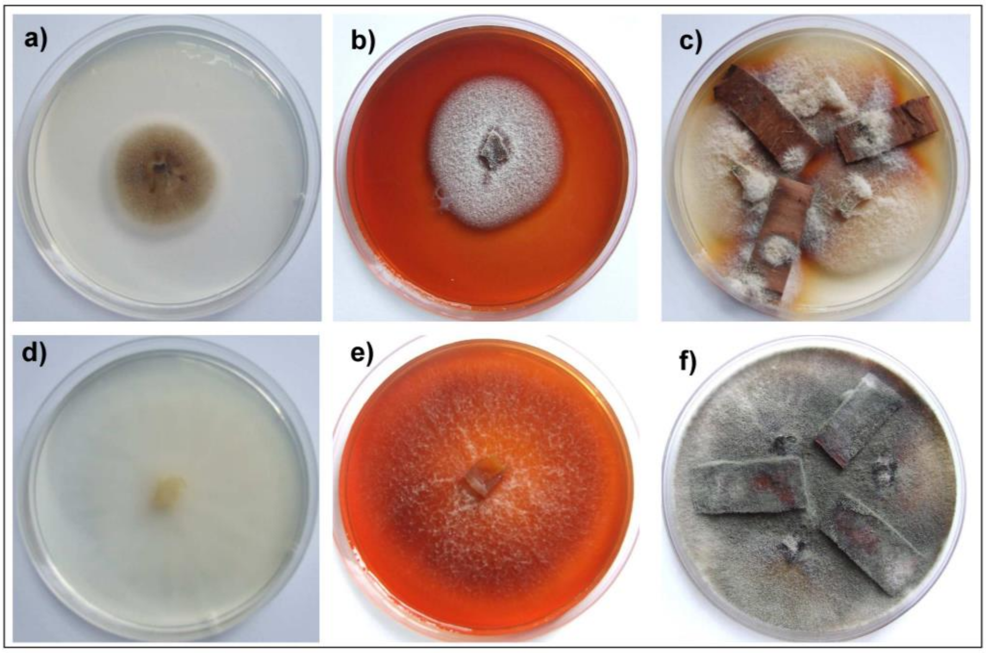

Figure S5. *Ceratocystis polonica* colonies after 6 days growth on: a) MEA medium, b) mineral medium without glucose and with phloem-extract medium, and c) mineral medium with phloem. *Ophiostoma penicillatum* (syn. *Grosmannia penicillata*) colonies after 6 days growth on: d) MEA medium, e) mineral medium without glucose and with phloem-extract medium, and f) mineral medium with phloem.
